# Comparison of the Compression and Tensile Modulus of Two Chosen Resins Used in Dentistry for 3D Printing

**DOI:** 10.3390/ma15248956

**Published:** 2022-12-15

**Authors:** Anna Paradowska-Stolarz, Andrzej Malysa, Marcin Mikulewicz

**Affiliations:** 1 Division of Dentofacial Anomalies, Department of Orthodontics and Dentofacial Orthopedics, Wrocław Medical University, Krakowska 26, 50-425 Wrocław, Poland; 2 Department of Experimental Dentistry, Wrocław Medical University, Krakowska 26, 50-425 Wrocław, Poland

**Keywords:** 3D-print, light-cured, resin, dentistry, synthesized system

## Abstract

(1) The CAD/CAM technique exploiting 3D printing is becoming more and more popular in dentistry. The resins are used in all the dental specialties, including conservative dentistry, prosthodontics, surgery, and orthodontics. The interest in investigating the different properties of dental materials has been an aim of researchers. The purpose of the presented study was to compare the properties of two 3D-printable dental resins (both rigid, used for medical purposes). (2) Methods: Ten blocks of two-type shapes were printed on a printer designed for medical use. The tensile modulus and compression were investigated and compared. The axial compression test was performed according to the PN-EN ISO 604:2003 norm, while the tensile test was performed according to the PN-En ISO 527-1-2019 (E) norm. In the first test, the sample size of the perpendicular shape was 10 ± 0.2 mm × 10 ± 0.2 mm × 4 ± 0.2 mm and in the second it was 75 mm, the end width 10 mm, and the thickness 2 mm. (3) Results: The statistical analysis based on ANOVA tests showed that all the obtained results were statistically significant. Both of the examined materials had similar properties and were resistant and stable in shape. The tensile modulus and compression tests performed on them gave similar results. They also showed high durability to compression and tensility. (4) Conclusions: Both of the examined materials were durable and rigid materials. BioMed Amber was more resistant to compression, while Dental LT clear was more resistant in the tensility test. Although both resins had similar physical properties, it is still disputable whether the chosen materials could be used interchangeably.

## 1. Introduction

Three-dimensional (3D) printing is a novel technique that is finding more and more use in the world today. In dentistry it is used mainly for dental restorations and prosthetic appliances, but it could also be applied for the printing of surgical specimens, such as surgical guides, custom parts, and anatomical models. The accuracy of the model allows for the precise preparation of the piece. Unfortunately, the scan and additional prints greatly increase the costs of the whole procedure; therefore, they cannot be used in all cases [1,2]. Among many advantages and better properties of printable materials, when compared to traditional ones, high accuracy, perfect shape molding, and fast print performance can be noted [2,3]. Regardless of its use, they should be characterized with high biocompatibility and non-irritating qualities [4]. For all purposes, 3D printing materials require 3D treatment planning, which involves the use of special customized programs and, in the case of surgery, CBCT to visualize the patient’s tissues [1]. STL files (standard tessellation language) files are used to produce the casts [5].

Three-dimensional printing has also been incorporated into medicine, including dentistry. The highest experience in this technology is in the domain of conservative dentistry and prosthetics. The printed pieces have been used to restore hard dental tissues for several years already. They are performed in two main production paths—AM (additive manufacturing) and MM (milling manufacturing). Both give comparable results, especially when considering polymers, in terms of mechanical properties [6]. Another novel perspective could be the use of hybrid materials, which would combine 3D printing with traditional techniques [7].

Additively manufactured pieces are commonly used to prepare interim crowns and other fixed dentures. Due to the lower parameters of hardness and fracture load than in MM techniques, they cannot support masticatory forces for a long period of time [6]. For removable dentures, PMMA (polymethylmetacrylate) is willingly used. In 3D printing technology, polymethylmetacrylate has been used for a long time and has properties very similar to the traditional ones [8,9,10].

Stereolitography (SLA) is the method for manufacturing pieces in the light-cure method. UV light is used to photocure the resin [11]. As mentioned before, 3D-printed materials have most use in prosthetic and restorative dentistry [1,2]. For this purpose, tooth-color resins are used [12]. The 3D print is also used in other branches of dentistry. They are a desired tool in surgery, including the placement of implants and mini-implants. The surgical guides produced with the use of 3D technology can also be used in orthognathic surgery, especially involving bimaxillary operations [13]. The operator should be aware, however, that drilling through the surgical guide induces heat of more than 42 °C and may lead to overheating of the surrounding tissues. This can lead to complications with healing, so the surgeon should be aware of this fact in planning [14].

The aim of this study was to present the differences in two rigid 3D-printable resins used in dentistry (BioMed Amber and Dental Clear LT by Formlabs). The second aim was to check whether the properties of the materials allow them to be used as substitutes. The null hypothesis for that study was that both resins presented in the study have the same properties.

## 2. Materials and Methods

Two 3D-printable resins designed for medical use—BioMed Amber (Amber UFI number E300-P0FU, Formlabs Ohio, Millbury OH, USA) and Dental Clear LT (LOT WY203N06, Vertex-Dental B.V., Soesterberg, The Netherlands)—were tested to evaluate their properties. The choice of those two resins was made by the authors and was based on the biomechanical and physicochemical properties similar to those of those materials, which was declared by the manufacturer. Both are biocompatible and are rigid materials.

According to the producers, BioMed Amber can be used to produce strong, rigid parts of medical devices. It can also be used to access surgical guides (also guides for cutting and drilling), models, functional threads, and sample collection kits. The properties of BioMed Amber are presented in Table 1.

According to the producers, Dental LT Clear can be used to manufacture individual appliances that require high esthetics and translucency. It is willingly used for fabrication of retainers, splints, occlusal guards, and can possibly be used as an aligner. The properties of Dental LT Clear resin are presented and compared with the BioMed Amber resin in Table 1.

**Figure 1:**
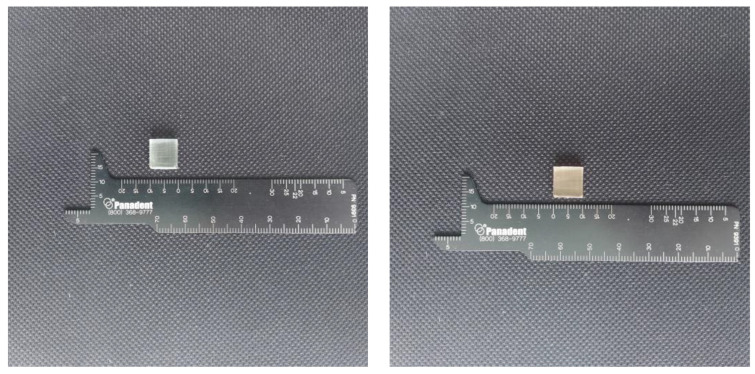
The shape of the samples used for the compression test, size of the sample: 10 ± 0.2 mm × 10 ± 0.2 mm × 4 ± 0.2 mm (**left**—Dental Clear LT, **right**—BioMed Amber).

**Figure 2:**
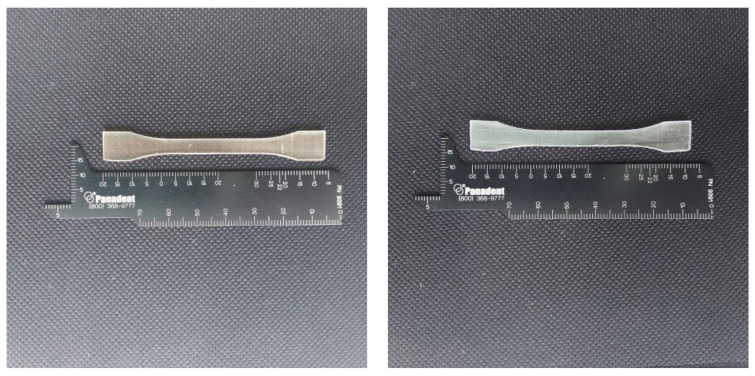
The dumbbell shape of the samples used for the tensile test, the sample size according to the ISO 527-2:2012 norm: length 75 mm, end width 10 mm, thickness 2 mm (**left**—BioMed Amber, **right**—Dental Clear LT).

The samples were prepared with use of a Formlabs Form 2 printer, that is a device calibrated for medical purposes. The samples were printed at a temperature of 35 °C. The layer thickness was 100 microns for both resins. After printing, the samples were rinsed in isopropyl alcohol for 10 min. Subsequently, another 10-min rinse was served. After being rinsed, the samples were dried for half an hour at room temperature. For the final hardening, a Form Cure specimen was used. The resins were originally packed, not used before, and opened just before the performance of the test. Two types of blocks were prepared for the test—a cube and dumbbell-shaped ones. For the ISO standards the minimal number of samples for that test is 5. In our test we used 10 samples of each shape [15]. The tests were performed with the use of the Universal Testing Machine Z10-X700 (AML Instruments, Lincoln, UK). The maximum moving grip of the device was 500 mm/min. The specimens that broke outside of the tested length area were deleted. Each measurement was made in five spots and was repeated three times each. After the test, compression and nominal strain were calculated. Figure 1 and Figure 2 present the shape of the examined samples.

The tensile strength was measured. For that purpose, dumbell specimens were prepared. The sizes of the samples were as follows: length 75 mm, width 10 mm, thickness 2 mm. After printing, the samples were air incubated for 24 h at 23 °C/50% RH. The measurements were performed three times in five spots for each specimen. The tensile test was performed with a speed of 5 mm/min. The force was measured at the breaking point. The specimens were measured in five places each. To reduce the risk of improper measurements, each was performed three times. 

Axial compression was measured according to the PN-EN ISO 604:2003 standard and the tensile test according to the PN-EN ISO 527-1:2019 (E). The samples of perpendicular sizes prepared for that test were 10 ± 0.2 mm × 10 ± 0.2 mm × 4 ± 0.2 mm. The samples were air incubated for 4 days in the temperature at a 23 °C/50% RH. Each sample was measured in width and thickness in five spots three times for each measurement. The compression test was performed with a speed of 1 mm/min. Compression and nominal strain were calculated on the basis of the following formula:

$$\begin{array}{cc} \mathrm{Compression} & \sigma \;=\;F:A\;[\mathrm{MPa}]\\ \mathrm{Nominal strain} & \varepsilon \;=\;\Delta L:L\;\times \;100\;[\%] \end{array}$$

where F—force (N), A—initial cross-sectional area measurement, L—distance between the compression plates (mm), ΔL—distance decrease (mm).

### Statistical Analysis

The Shapiro–Wilk test was used to choose which type of ANOVA test to use. With *p* > 0.05, a parametric ANOVA test was used. After qualification with the Shapiro–Wilk test, after consultation with the statistician, the authors decided to use a parametric test (t-Student) to compare the average values in compression and tension tests between Dental LT Clear and BioMed Amber.

All the measurements were made based on STATISTICA v. 13 program (TIBCO Software Inc., Palo Alto, CA, USA).

## 3. Results

**Figure 3:**
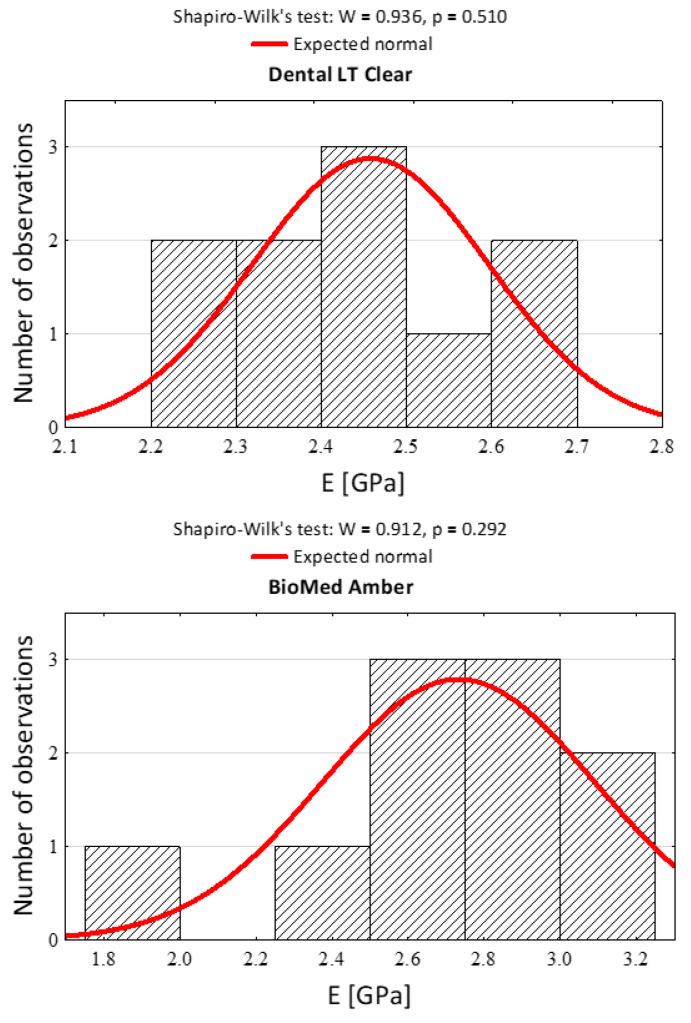
Histograms of the results of Young’s modulus in compression against the background of the normal distribution for three dental materials and the results of normality tests.

**Figure 4:**
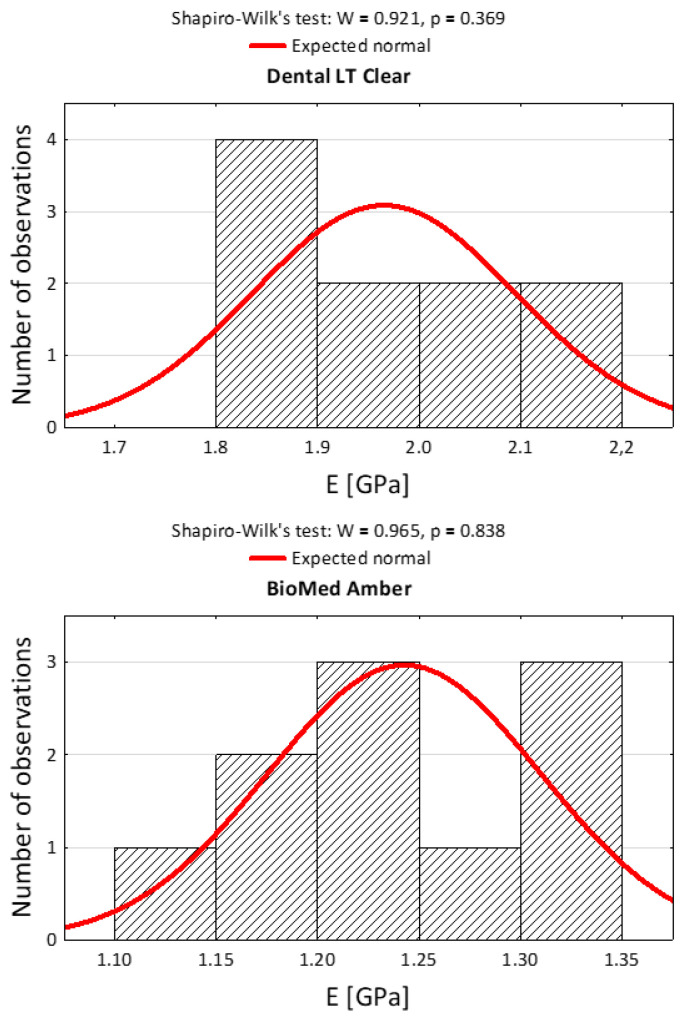
Histograms of the results of the measurement of Young’s modulus in tensile against the background of the normal distribution for the three dental materials and the results of the normality tests.

After the statistical qualification to the research and the choice of the appropriate statistical tests, histograms were prepared. The histograms in Figure 3 and Figure 4 present the statistical qualification of the research.

The distribution of the data helped us to decide to use the T-student test to evaluate further data. The basic descriptive statistic was presented in Table 2. The statistically significant *p*-values are presented in red.

**Figure 5:**
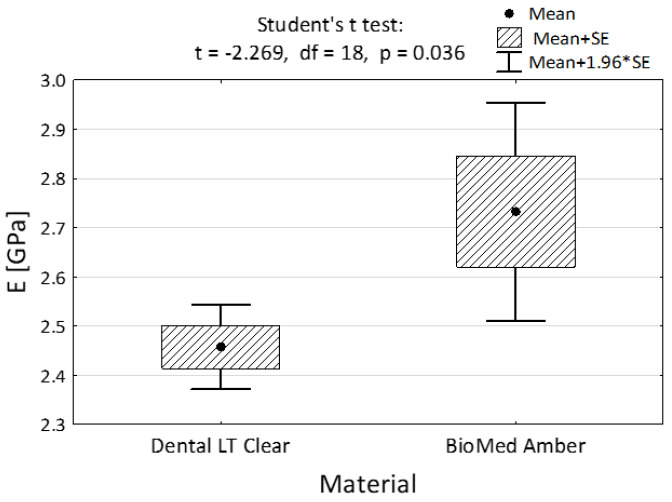
Compressive modulus of the two dental materials and the result of the Student’s *t*-test.

**Figure 6:**
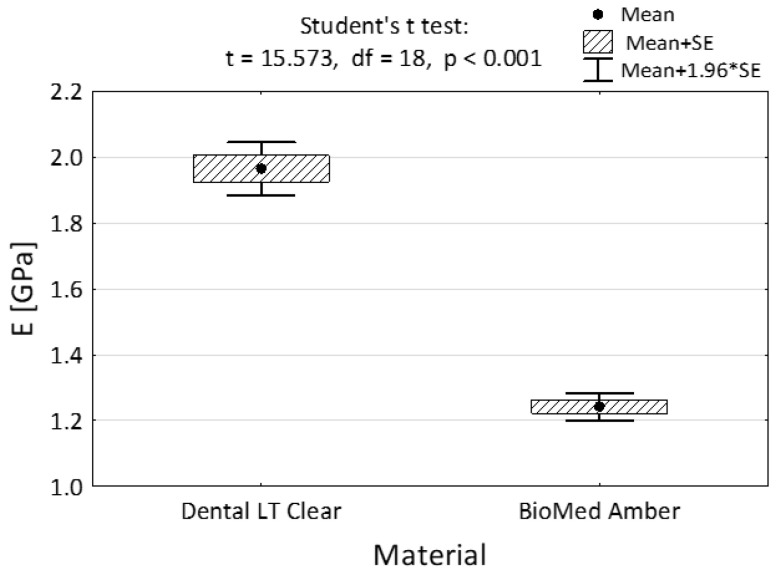
Tensile modulus of the two dental materials and the result of the Student’s *t*-test.

All the results in the average modulus of elasticity both in compression and in tension turned out to be statistically significant (2.46 GPa vs. 2.73 GPa—Figure 5 and 1.97 GPa vs. 12.24 GPA, Figure 6).

Both materials had stable and quite predictable properties in the tensile modulus and compression tests. BioMed Amber resin turned out to be more resistant to compression than Dental LT Clear, though it turned out to be destroyed earlier in the tensile test. The tests show that both of the materials were durable, stiff, and resistant to damage. This shows that the resins were stable in the physical properties presented. 

According to the results of the study, the null hypothesis was refuted.

## 4. Discussion

The perfect 3D printed object has a high accuracy and stability. It also should be characterized by the mechanical properties suitable for the application. Therefore, new resins are being investigated [16]. The other possibility is to search for possible other uses of the resins that are already in use. This type of research is a frequent manner of studies and is interesting in the authors’ opinion. It had been previously performed by the researchers on other dental materials, e.g., composites [17,18]. The investigated materials (BioMed Amber and Dental LT Clear), printed by Form2 printer from Formlabs, had the specified temperature, layer thickness, and other printing parameters in the chip that is built into the cartridge. However, it is also possible to use other types of resin with that printer [19]. Both of the investigated materials revealed a high accuracy and stability, which makes them good candidates to print 3D objects, especially in medicine, including dentistry. To the best of authors’ knowledge, this kind of comparative research is novel and had not been performed before by any researcher.

Dental LT Clear is most commonly used for the preparation of splints, especially in patients treated for temporomandibular disorders, including bruxism. As also shown in our study, when compared to the other ones, this is a durable and stiff material [20]. BioMed Amber is mainly used for the production of surgical guides, but as shown in our research, it has a similar rigidness and stiffness to Dental LT Clear, so it could be evaluated further for potential use as a splint. The main, visible difference between the two resins was that Dental LT Clear showed a high translucency and was highly esthetic, while as BioMed Amber had ayellowish glow in the transparent basis. Both materials showed a high resistance to compression and tensility. Although, as proven in the research, the chosen materials had similar physical properties, they are used for other purposes. Further research should be performed to evaluate whether they could be used interchangeably. Probably due to the yellow color, BioMed Amber could not be used as an orthodontic aligner, although the development of 3D printed aligners is high—they are more durable and accurate than the thermoformed ones, which have willingly been used previously [21]. Therefore, as shown in our research, Dental Clear LT seems to be a perfect candidate for that purpose. It is disputable whether the materials can be used as substitutes, especially when biocompatibility has been investigated [22]. CAD/CAM resins might release monomers that could be irritating to tissues, although it seems to be an acceptable dose, unless there is an allergy to the monomer [23,24,25].

Modern orthodontics is based on the reduction in time that a patient spends in the dental chair. The first modification was the incorporation of self-ligating brackets into the treatment. They allowed a shorter period of time on the dental chair, as well as rarer orthodontic check-ups [26,27]. Incorporation of clear aligners to the orthodontic treatment allowed the chair time to be reduced as well [28]. Customised appliances are the other type of chair-time reduction, because they require rarer dental visits, especially when additional appliances such as Benesliger are added [29]. As 3D printed materials are also more commonly used for orthodontic purposes, such as customized appliances, the perspective is that they could be used 24 h a day. Due to this long-term use during the day and the lack of possibility of washing the teeth after each meal, they could be exposed to beverages and food. Dental Clear LT is a material approved for long-term use in contact with tissues, so RME (rapid maxillary expander) could be produced from this resin [30]. Due to permanent exposure to food and saliva, the properties of those materials might change. Surface fractal evaluation showed that the materials used for that purpose are not resistant to the damaging factors present in food and drinks—the novel study carried out revealed that dental aligners are sensitive to reaction to beverages such as coca cola and orange juice [31]. Investigating for fractal dimension and texture analysis, to the authors’ mind, is an interesting trend in dentistry. The authors of this research also think that in relation to Dental Clear LT, further research could be performed on that topic. That could help us compare this resin to traditional thermoformed plates commonly used in dentistry, for example, as Essix retainers [32].

## 5. Conclusions

The performed study allowed us to form several conclusions. 

Both materials had similar physical properties. However, the statistical evaluation revealed some differences. Both materials were resistant to compression and tensility tests—they are rigid and durable materials. The study we carried out showed that both materials were rigid and resistant to compression and tensility. Dental LT Clear was less resistant to compression than BioMed Amber. BioMed Amber was less resistant to the tensility test. It is an interesting fact that those two materials differ in that manner and should be investigated further. The presented study could be used as a basis for investigation of further studies on the printable dental materials.

## 6. Limitations

The paper, although prepared to the best of the authors knowledge, has some limitations that should be pointed out. The first is that the chosen resins were from one company only. Although the authors tried to choose the most similar resins according to their properties, to our mind the choice could still be a subjective choice. Other companies may produce similar resins that were not included in our study. Moreover, although the performed study complied with ISO standards, the number of the samples needs to be extended in the future.

## Figures and Tables

**Table 1:** Comparison of the properties of BioMed Amber and Dental LT Clear Resins.

	BioMed Amber	Dental LT Clear
Characteristics	- Biocompatible - Suitable for sterilization and disinfection	- Biocompatible - Not suitable for sterilization - Highly esthetic
Physical properties	- Rigid - Strong - Transparent, beige	- Rigid - Strong - Transparent, translucent
Skin and mucosa	- Suitable for short-term skin and mucosal contact	- Suitable for skin and mucosal contact
ISO standards	ISO 13485 EN ISO 10993-5:2009 ISO 10993-10:2010/(R)2014 ISO 10993-10:2010/(R)2014	EN-ISO 10993-1:2009/AC:2010 EN-ISO 10993-3:2009 EN-ISO 10993-5:2009 EN 908:2008
Potential use in dentistry	- Strong, rigid parts of medical devices - Surgical guides - Morels - Functional threads - Sample collection kits	- Individual appliances with high esthetics and translucency - Retainers - Splints - Occlusal guards - Aligners
Chemical components	7,7,9(or 7,9,9)-trimethyl-4,13-dioxo-3,14-dioxa-5,12-diazahexadecane-1,16-diyl bismethacrylate 2-hydroxyethyl methacrylate Phenyl bis(2,4,6-trimethylbenzoyl)-phosphine oxide	7,7,9(or 7,9,9)-trimethyl-4,13-dioxo-3,14-dioxa-5,12-diazahexadecane-1,16-diyl bismethacrylate 2-hydroxyethyl methacrylate (Note D) Reaction mass of Bis(1,2,2,6,6-pentamethyl-4-piperidyl) sebacate and Methyl 1,2,2,6,6-pentamethyl-4-piperidyl sebacate diphenyl(2,4,6- trimethylbenzoyl)phosphine oxide Acrylic acid, monoester with propane-1,2-diol ethylene dimethacrylate 2-hydroxyethyl acrylate mequinol, 4-methoxyphenol, hydroquinone monomethyl ether

**Table 2:** Basic statistics of the mechanical properties of the dental materials (Dental LT Clear and BioMed Amber).

Young’s Modulus (GPa)	Material		*p*-Value
	Dental LT Clear	BioMed Amber	
	N = 10	N = 10	
Compression			0.036
Mean ± SD	2.46 ± 0.14	2.73 ± 0.36	
Min–Max	2.26–2.68	1.92–3.17	
Tensile			<0.001
Mean ± SD	1.97 ± 0.13	1.24 ± 0.07	
Min–Max	1.80–2.15	1.14–1.34	

## Data Availability

The datasets generated and/or analyzed during the study are available from the corresponding author on reasonable request.
